# Photonic-plasmonic mode coupling in nanopillar Ge-on-Si PIN photodiodes

**DOI:** 10.1038/s41598-021-85012-z

**Published:** 2021-03-11

**Authors:** Lion Augel, Jon Schlipf, Sergej Bullert, Sebastian Bürzele, Jörg Schulze, Inga A. Fischer

**Affiliations:** 1 Micro and Nano Systems, Brandenburg University of Technology Cottbus-Senftenberg, 03046 Cottbus, Germany; 2 Institute of Semiconductor Engineering, University of Stuttgart, 70569 Stuttgart, Germany; 3 Experimental Physics and Functional Materials, Brandenburg University of Technology Cottbus-Senftenberg, 03046 Cottbus, Germany

**Keywords:** Nanoscale devices, Electrical and electronic engineering, Optoelectronic devices and components

## Abstract

Incorporating group IV photonic nanostructures within active top-illuminated photonic devices often requires light-transmissive contact schemes. In this context, plasmonic nanoapertures in metallic films can not only be realized using CMOS compatible metals and processes, they can also serve to influence the wavelength-dependent device responsivities. Here, we investigate crescent-shaped nanoapertures in close proximity to Ge-on-Si PIN nanopillar photodetectors both in simulation and experiment. In our geometries, the absorption within the devices is mainly shaped by the absorption characteristics of the vertical semiconductor nanopillar structures (leaky waveguide modes). The plasmonic resonances can be used to influence how incident light couples into the leaky modes within the nanopillars. Our results can serve as a starting point to selectively tune our device geometries for applications in spectroscopy or refractive index sensing.

## Introduction

Metal nanostructures that support localized surface plasmons (LSP), i.e. collective excitations of the free electron gas within the nanostructures, enable local electromagnetic field enhancement at the nanoscale^1^. This has seen wide applications ranging from biosensing^2,3^, in which the sensitivity of the LSP properties to changes in the refractive index induced by analyte-ligand binding events is exploited, and wavelength-selective absorption enhancement in solar cells^4^, bulk detectors^5^ as well as nanoscale photodetectors^6^. LSPs can be utilized to focus incident light into nanoscale semiconductor volumes. In these applications, the subwavelength-sized semiconductor structures themselves can sustain discrete photonic modes^7,8^ at selected excitation wavelengths, so-called “leaky waveguide modes”, which enable spectral filtering of absorbed radiation and can pave the way for future integrated light sources^9,10^. Photodetectors using nanowires and nanopillars as two dimensional nanostructures have been shown to exhibit improved absorption efficiency through an increased surface-to-volume ratio^11–15^ as well as fast signal detection^16^. Furthermore, semiconductor nanostructures are also beneficial for integrating non-silicon active materials in complementary metal oxide semiconductor (CMOS) processes on Si substrates: by reducing the active volume of materials such as III–V compounds^17^ or Ge the impact of dislocations on dark current paths can e.g. be reduced.

Combining plasmonic and photonic nanostructures can be a path towards realizing efficient nanoscale photodetectors for applications in high-speed photonic data transmission, spectroscopy or sensing. However, the full potential of this approach can only be realized if the interplay of plasmonic and photonic modes can be tuned. Furthermore, a robust fabrication process is required for reliable CMOS integration. Large-scale CMOS production relies on top-down manufacturing processes as they ensure reproducibility and thereby prevent device behavior variations. However, contacting active nanophotonic devices in planar setups such as focal plane arrays is technologically challenging as the contact material has to be transparent, besides exhibiting a high conductivity at low contact resistivity. Here, the standard contact metal layers are too thick to be transparent, while transparent conductive oxides (TCO) often suffer from increased contact resistance^18^ and the integration of Graphene layers as transparent contact materials into CMOS processes is still challenging^19^.

Plasmonic nanoapertures (NA) in thin metal layers offer a means to reliably contact nanophotonic devices while simultaneously combining photonic and plasmonic nanostructures in a self-aligned fabrication process^6,20–22^. Vertical nanowires can be contacted by evaporating metal onto the sample under an angle in such a way that the exposed parts of the nanopillars (NP) act as a shadow mask and enable the formation of NA adjacent to the semiconductor NP. Prior research efforts in this area were e.g. focused on investigating the role of extended surface plasmon polariton Bloch waves in arrays of III-V NP photodiodes. Surface plasmon polariton Bloch waves allow to funnel radiation through optically dense metallic films by extraordinary transmission^23^. Furthermore, the interplay of local surface plasmon resonances (LSPR) excited in the metal cap with the semiconductor NP was shown to enable photodetection at photon energies below the semiconductor bandgap^21^.

**Figure 1:**
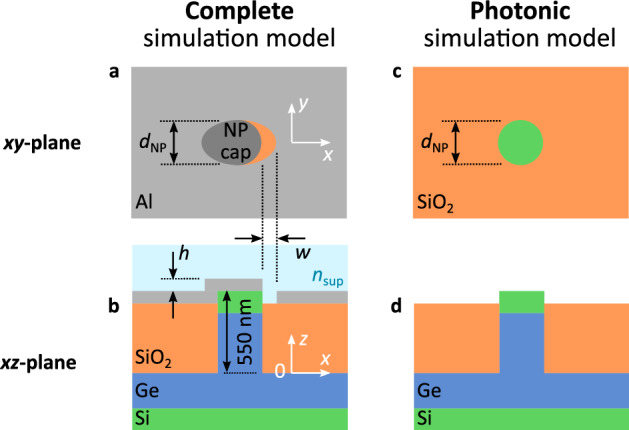
Sketch of the (**a**), (**b**) complete simulation setup used to model the NP-PD and (**c**), (**d**) simplified photonic simulation model without metallization layer, Layer structures as well as structural parameters used throughout this work are indicated in the top view (**a**), (**c**) and cross-sectional images (**b**), (**d**).

Here, we investigate the interplay of LSPR and photonic modes in a CMOS compatible device, in which a crescent shaped NA within the aluminum contact metallization is formed in proximity to a vertical NP Ge-on-Si PIN photodetector (NP-PD, Fig. 1a, b). Ge is widely used as a photodetector material in foundry processes. In contrast to previous research efforts we focus on a device geometry in which device responsivity is strongly influenced by the interplay of localized plasmonic excitations in the crescent-shaped NA with photonic modes in the NP-PD. For our devices, the most relevant geometry parameter is the diameter of the NP *d*_NP_ as it allows to tune both the leaky waveguide modes as well as the LSPR. We, therefore, discuss the optoelectronic device properties as a function of diameter as well as incident wavelength based on simulation and experimental data. Furthermore, we show how the interplay between plasmonic and photonic modes can be tuned by adjusting geometry and material parameters.

## NP Ge-on-Si PIN photodetector: simulation results and experimental verification

Our theoretical and experimental investigation is focused on a device geometry (Fig. 1a, b) that enables photodetector operation at wavelengths up to λ ∽ 1550 nm. This range is of technological interest for optical data transmission up to the C band. Energies below 0.8 eV or wavelengths longer than 1550 nm, respectively, are cut off by the bandgap of Ge.

In order to investigate the interaction between the photonic modes of the NP and the plasmonic modes of the NA, we first discuss the optical properties of the NP separately based on simulation results obtained using Lumerical’s finite-difference time-domain (FDTD) solutions package as well as analytical calculations.

The absorption characteristic of the NP-PD (Fig. 1c, d) can be expected to be dominated by leaky waveguide modes, which, for each diameter *d*_NP_, lead to absorption maxima at certain wavelengths of the incident light^15^. For a Ge wire of infinite length in direction ***l*** and diameter *d* that is illuminated perpendicularly to the wire’s axis (free space wave vector ***k***_0_⟂***l***), these modes can be determined analytically. Depending on the orientation of the electric field vector of the incident light w.r.t. the wire axis, transverse magnetic modes TM_*ml*_, where ***H***⟂***l***, or transverse electric modes TE_*ml*_, where ***E***⟂***l***, can be excited within the wire. Their mode profiles are specified by an azimuthal mode number *m* and a radial mode number *l*. The TM-modes can be found by solving^24^

1 $$ \frac{{n_{{{\text{Ge}}}} \left( \lambda \right)J_{m}^{\prime } \left( {n_{{{\text{Ge}}}} \left( \lambda \right)k_{0} d} \right)}}{{J_{m} \left( {n_{{{\text{Ge}}}} \left( \lambda \right)k_{0} d} \right)}} = \frac{{H_{m}^{\prime } \left( {k_{0} d} \right)}}{{H_{m} \left( {k_{0} d} \right)}}, $$

Here, *J*_*m*_ and *J*′_*m*_ (*H*_*m*_ and *H*′_*m*_) denote the Bessel (Hankel) function of the first kind and *m*-th order and its derivative, respectively. Furthermore, in the derivation of Eq. 1 it is assumed that the Ge wire is surrounded by air^24^. In the following, we restrict our discussion to the TM-modes since the peak positions of the TE-modes coincide with those of the TM-modes for Ge wires of small diameter^24^—which should not imply that the exact modes *ml* match each other.

**Figure 2:**
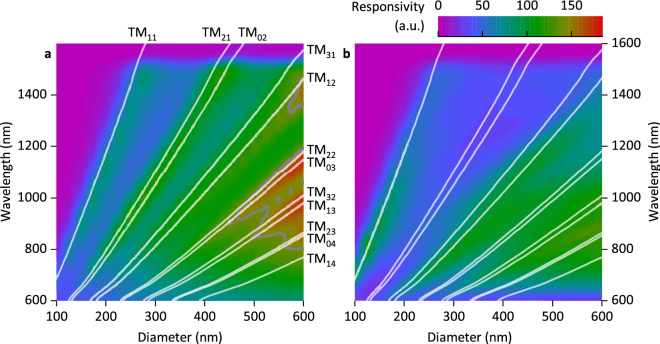
Simulated spectral responsivity (see Eq. 2 in the methods chapter) as a function of the diameter *d*_NP_ of the NP-PD without metallization (Fig. 1c, d). The lines show the results obtained from solving Eq. 1. The illumination was chosen to be (**a**) parallel (***E***_0_‖***n***,* k*_0_⟂***n***) and (**b**) normal (***E***_0_⟂***n***,* k*_0_‖***n***) to the substrate plane (***n*** is the normal vector of the substrate).

In order to understand the predictive power of the analytical calculation for our NP photodetectors of finite height, we compare the resulting modes with simulation results obtained from FDTD calculations, in which the actual NP geometry is taken into account, i.e. a NP height of 550 nm is assumed and the NP is embedded within SiO_2_ up to a height of 500 nm. In the analytic calculation, the incident wave is assumed to impinge perpendicularly to the NP axis. Using the reduced simulation setup without metallization (photonic simulation setup, Fig. 1c, d) reveals that the absorption peaks within the Ge layer of the NP are predicted by the analytic model in a close to perfect agreement as long as the vector of the incident radiation is in plane with the NP substrate (Fig. 2a, solid lines). The assumption of an infinite nanowire in vacuum is therefore well suited to describe the optical properties of the finite NP even though it is mostly embedded in SiO_2_. Orienting the source above the photonic simulation model (illumination direction perpendicular to the NP substrate) reveals that some of the expected modes can no longer be directly excited by the impinging radiation (Fig. 2b). Additionally, this gives rise to hybrid modes which are no longer TM or TE due to the relative orientation of wire axis and wave vector^15^.

**Figure 3:**
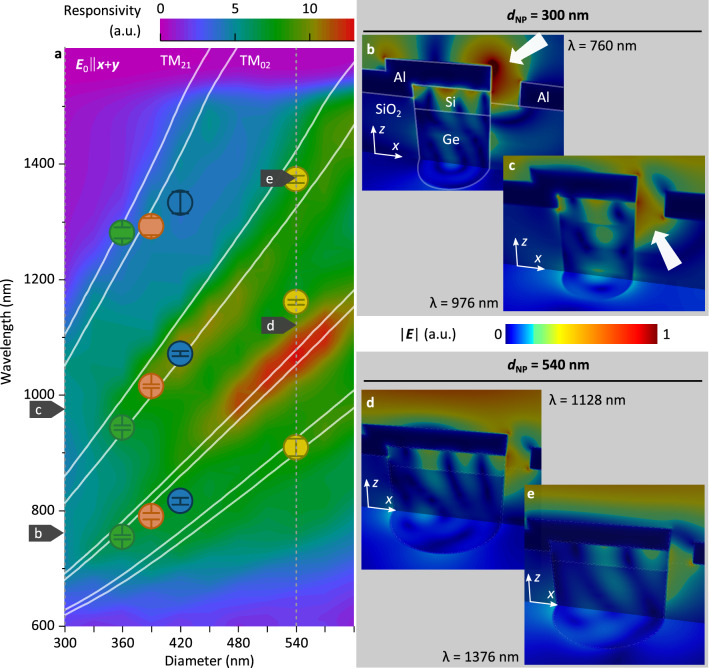
(**a**) Simulated spectral responsivity as a function of the NP diameter *d*_NP_ for a complete device as shown in Fig. 1. The overlay shows the photonic modes as described by Eq. 1. The points indicate the maxima of the Gaussian functions used to fit the measured responsivities shown in Fig. 5. The orientation of the incident field was chosen to be ***E***_0_‖(***x*** + ***y***). (**b**)–(**e**) Cross-sectional images of the absolute value of the electric field distribution inside NP-PDs with *d*_NP_ = 300 nm at incident wavelengths of λ = 760 nm (**b**) and λ = 976 nm (**c**) (corresponding to absorption maxima, see Fig. 6a for *n* = 1.0) and NP-PDs with *d*_NP_ = 540 nm at λ = 1128 nm (**d**) and λ = 1376 nm (**e**). The orientation of the incident field for (**b**)-(**e**) is ***E***_0_‖***x***. The horizontal plane in (**b**)–(**e**) is approximately 250 nm below the top Si-Ge interface.

Next, we investigate the optical properties of the combined setup, i.e. for a photodetector geometry in which the effects of the NA and the NP are combined (complete simulation model, Fig. 1a, b, with *w* = 100 nm, *h* = 50 nm and a pillar height of 550 nm, ***E***_0_‖(***x*** + ***y***)). Simulation results for the responsivity of the devices under vertical illumination show a mode structure that, again, can be matched with analytical results for TM-modes (Fig. 3a, solid lines). Thus, a first consequence of combining the plasmonic (NA) and photonic (NP) nanostructures is that radiation which impinges perpendicularly onto the device is funneled into the leaky modes of the NP by the NA: The NA scatters the incoming radiation and thereby alters the electric field vector distribution, which enables the excitation of additional modes (particularly the modes TM_02_ and TM_21_) compared to NP under vertical illumination in the absence of the NA (Fig. 2b).

This can be further illustrated by investigating cross sections of the absolute electric field distribution $\left| {{\varvec{E}}\left( {x,y,z} \right)} \right|$ for two selected devices with *d*_NP_ = 300 nm (Fig. 3b, c) and *d*_NP_ = 540 nm (Fig. 3d, e) under illumination with ***E***_0_‖***x***. For the device with *d*_NP_ = 300 nm and an illumination wavelength of λ = 760 nm (Fig. 3b) a region of enhanced electric field intensity (white arrow) is located on top of the NP cap. Increasing the illumination wavelength to λ = 976 nm shifts this region of enhanced electric field intensity so that it is now mainly located within the NA (white arrow, Fig. 3c). The highest responsivity is predicted for devices with *d*_NP_ = 540 nm at an illumination wavelength λ = 1128 nm (Fig. 3a). While regions of field enhancement can also be seen at the interface between semiconductor and metal cap on top of the NP, the regions of strongest electric field intensity are situated within the NA (Fig. 3d). Increasing the illumination wavelength to λ = 1376 nm still leads to a (weaker) enhancement of electric field intensity within the NA (Fig. 3e). One possible explanation for the region of highest responsivities (for 480 nm ≤ *d*_NP_ ≤ 580 nm and 950 nm ≤ λ ≤ 1150 nm) is, thus, that for these geometries and incident wavelengths, the funneling of radiation by the LSPR modes within the NA into the leaky modes of the NP is particularly efficient. An unambiguous identification of the leaky modes in the plots of the electric field intensities is difficult as the limited size of the Ge layer, the Si cap and the metallization can be seen to influence the shape of the modes (Fig. 3b–e). We find that the analytic model (Eq. 1) provides a helpful indication of the operation range of such devices as simulated regions of high responsivity and analytic modes are in good agreement (Fig. 3a).

**Figure 4:**
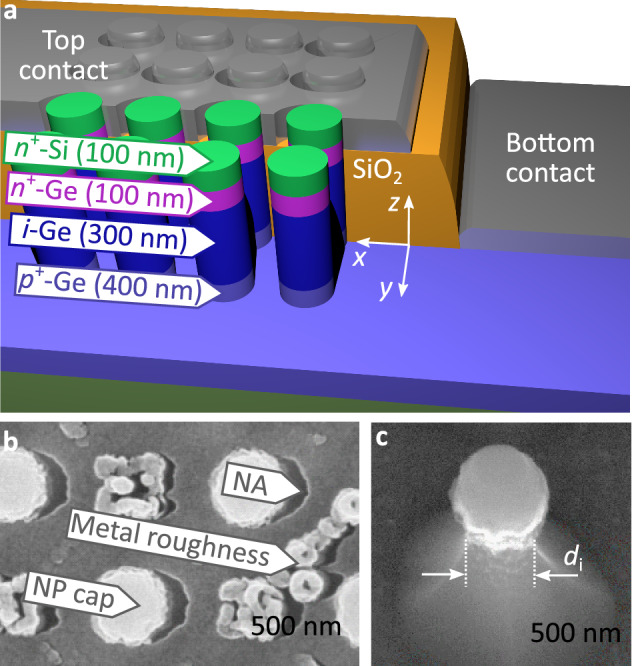
(**a**) 3D sketch of the NP-PD device. Metallization and passivation are partly removed. (**b**) Scanning electron microscopy (SEM) top view image of the device. (**c**) SEM image of a single NP after etching and before hardmask removal.

Prior to investigating the coupling of plasmonic and photonic modes in more detail we discuss experimental results that serve to support our simulation results. For that, NP-PD devices were fabricated in a CMOS compatible process (Fig. 4a, see method chapter for details). While we discuss single NP-PD in simulation, our devices were fabricated as regular arrays of NP-PD in which the plasmonic and photonic excitations can, in principle, be modified by arranging the individual devices in an ordered lattice. In our case, however, a large distance between individual NPs as well as a high metal layer roughness effectively suppress photonic crystal modes^15^ as well as plasmon polariton Bloch waves, respectively (Fig. 4b). This enables us to measure single-device characteristics in an array configuration in which the total photocurrent is significantly enhanced, but it also introduces a broadening in the measured spectra as a result of geometry variations introduced by the fabrication process.

**Figure 5:**
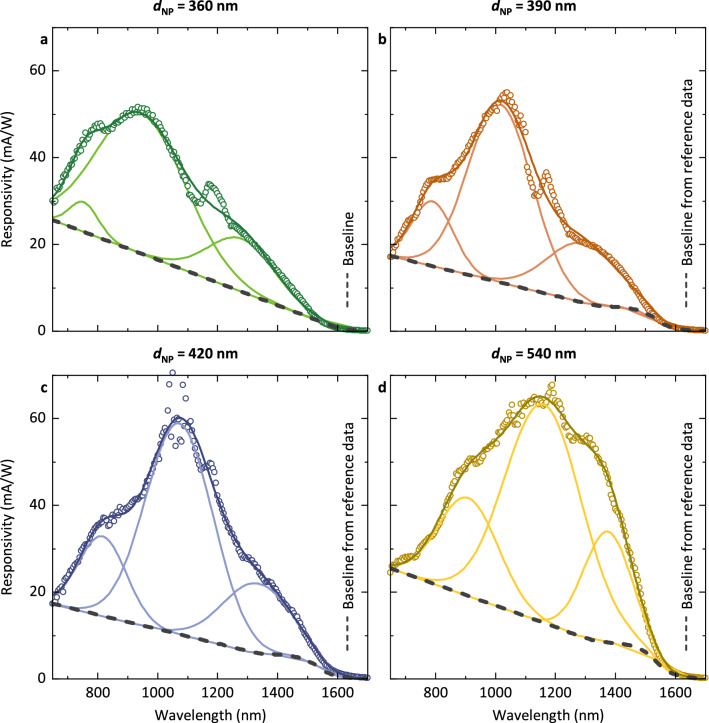
Measured spectral responsivity (○) of NP-PD devices with varying diameter *d*_NP_. For each measurement a peak analysis with three Gaussian functions (–) was carried out. The peak positions and corresponding errors are indicated in Fig. 3a. The baseline (‐ ‐ ‐) incorporates a reference responsivity spectrum obtained from a bulk photodetector with an identical semiconductor layer stack (see Fig. S7) and was adjusted for each measurement to take into account process variations. The peak at λ = 1180 nm is an artifact originating from the light source (wavelength of the pumping laser).

The measured spectral responsivity curves of devices with different diameters can be fitted by three Gaussian functions (Fig. 5). The extracted peak positions are also plotted in Fig. 3a. We note that in our devices, the NP diameter *d*_i_ varies over the device height as a result of the etching process (Fig. 4c). In particular, underetching of the Ge layer w.r.t. the Si cap layer can be observed. This results in an error when determining the diameter *d*_NP_ based on top view SEM images. Hence, the final assignment was done by comparing simulated and measured responsivity spectra with an error of ± 5 nm. Nonetheless we find that the extracted peak positions are in good agreement with the simulation and the analytical calculations, which provides experimental verification of our simulation results. Finally, we also note that the extracted series resistance of our devices was found to be in the order of 10 Ω (Supplementary Information, Fig. S5), which shows that our metal contacts can indeed serve as low-resistance contacts to integrated photonic devices.

## Influence of geometry and material parameters

In order to obtain a more quantitative understanding of the interplay of plasmonic and photonic modes, we selectively varied geometry and material parameters of our NP-PD in simulation. This discussion does not claim to be exhaustive, it can rather serve as a starting point for further investigations. Here, we chose to vary the superstrate refractive index *n*_sup_, width *w* of the NA, and the height difference *h* between the top of the NP and the SiO_2_ surface in which the NP is embedded. All of these parameters mainly influence the NA LSPR properties while leaving the NP leaky mode spectrum largely unchanged, which serves to provide insights into the coupling of plasmonic and photonic modes.

In the crescent-shaped NA plasmonic resonances can be (resonantly) excited under vertical illumination depending on the polarization of the incoming light, i.e. ***E***_0_‖***x*** and ***E***_0_‖***y*** (Fig. 1a)*,* where ***E***_0_ is the electrical field vector. For a single aperture, we can expect to observe a Gaussian transmission profile, whose spectral position and peak height depends on the length and width of the aperture, the refractive index of the surrounding dielectric as well as the polarization of the incident light^1^. Here, we therefore discuss the behavior for the two orientations of the polarization of the incoming light separately.

**Figure 6:**
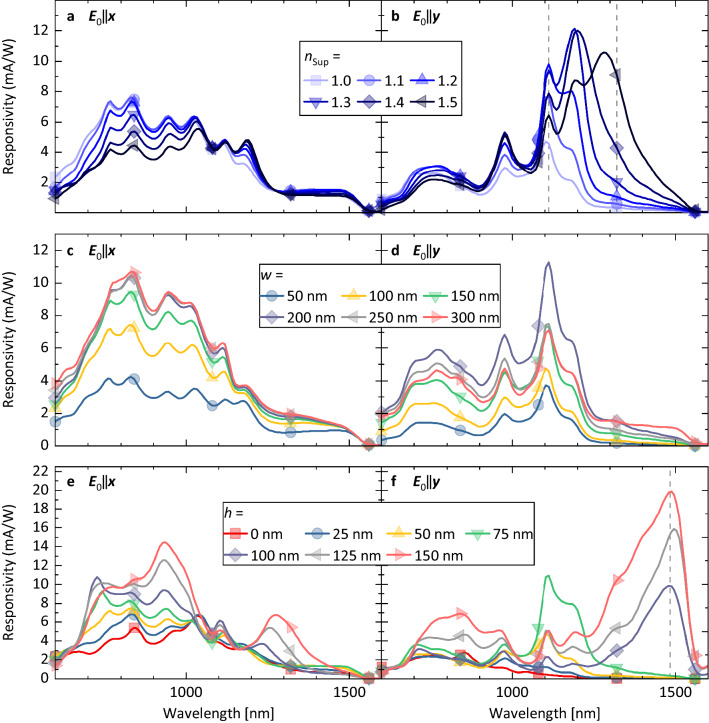
Simulated responsivities of a NP-PD device with a diameter of *d*_NP_ = 300 nm (Fig. 1a, b) and illuminated with ***E***_0_‖***x*** and ***E***_0_‖***y*** using varying material and geometry parameters. Results are shown for (**a**) and (**b**) variations of the superstrate refractive index *n*_*sup*_ at *w* = 100 nm and *h* = 50 nm, (**c**) and (**d**) variations of the NA geometry parameter *w* at *h* = 50 nm and *n*_*sup*_ = 1.0, (**e**) and (**f**) variations of the geometry parameter *h* at *w* = 100 nm and *n*_*sup*_ = 1.0. Field profiles ***E***(x,y,z) for wavelengths indicated by the dashed lines are shown in the supplementary information.

Increasing the refractive index of the superstrate above the NP-PD (*d*_NP_ = 300 nm, *w* = 100 nm and *h* = 50 nm) can be expected to shift the peak position of the NA LSPR to larger wavelengths. This provides an efficient means of shifting the LSPR peak with respect to the leaky modes. Indeed, simulation shows that increasing *n*_*sup*_ can be seen to strongly influence responsivity for incident wavelengths 1200 ≤ λ ≤ 1500 nm for ***E***_0_‖***y*** by altering the relative peak intensities (Fig. 6b).

Varying the width *w* of the NA while keeping the parameters *d*_NP_ = 300 nm and *h* = 50 nm fixed only weakly influences the peak positions and their relative intensities (Fig. 6c, d). For ***E***_0_‖***x*** an increase in *w* results in an overall responsivity enhancement resulting from the increase in area through which light can be transmitted into the devices, this effect saturates at *w* = 200 nm. For ***E***_0_‖***y*** the maximum responsivity is attained at 200 nm: further increasing *w* can be seen to reduce the efficiency of light coupling into the NP.

Varying *h* can be seen to have the strongest wavelength-dependent effect on device responsivity: In particular, the transition between *h* = 75 nm and *h* = 100 nm induces a marked change in the responsivity spectra for both polarization directions, with additional peaks appearing in the spectra at λ > 1200 nm. At *h* = 100 nm the lower Al/ SiO_2_ interface is at the same height as the upper n-Ge/ i-Ge interface. For all devices with *h* ≥ 100 nm the evanescent fields of the LSPR can directly penetrate into the i-Ge and thereby significantly increase the electric field strength (and, as a consequences, improve the generation of a photocurrent) within the device (see supplementary information for field intensity plots). For ***E***_0_‖***y*** with its stronger cavity fields this effect is more pronounced. Here, we attribute the intensity shift to a strong peak at λ = 1483 nm for *h* ≥ 100 nm to the excitation of a different leaky mode (comparable with shifting from TM_21_/TM_02_ to TM_11_ for the nanowire alone, see Fig. 2) in combination with the direct bandgap of Ge acting as a cutoff for responsivity spectra at λ ∽ 1550 nm.

## Conclusion

We investigated Ge-on-Si NP-PD in combination with plasmonic NA resulting from a self-aligned contact fabrication process. Our CMOS compatible device fabrication process can be used to establish low-resistivity contacts with < 10 Ω to Ge-on-Si PIN NP-PD while keeping the contact permeable for illumination through crescent-shaped nanoapertures located next to each NP-PD. Furthermore, both our simulation and experimental results show that the interplay of photonic modes in the semiconductor nanostructures with plasmonic modes in the NA can be utilized to shape the wavelength-dependent absorption characteristic of the devices. While the absorption characteristic of the NP-PD device is largely determined by the diameter-dependent leaky waveguide modes, which lead to absorption peaks at specific wavelengths within the Ge layer of the photodetector, the NA can be used to tune how light couples into these modes.

A simple analytic model based on an infinite Ge wire offers a surprisingly reliable way to predict device absorption characteristics qualitatively. Relevant device diameters for near-infrared radiation up the C band (1550 nm) range from 300 to 600 nm. We investigated several geometry and material parameters that enable tuning the plasmonic resonances separately from the photonic modes in order to explore the interplay of photonic and plasmonic resonances in more detail. We found that changing the superstrate refractive index as well as changing the height of the NP protruding from the dielectric in particular can serve as a means to tune the NA properties independently of the NP properties in order to enhance absorption at selected wavelengths. The index variation can e.g. be achieved by depositing an additional passivation layer (SiO_2_) in the device fabrication process.

Our work can serve as a starting point for further development of Ge-on-Si NP-PD for fast device operation and wavelength-selective photocurrent generation, e.g. selectively improving device characteristics for operation at telecom wavelengths. Furthermore, it would be interesting to explore the potential of such a device for a robust and compact integrated refractive index biosensor that is aimed at detecting very small sample volumes.

## Methods

The simulations were carried out using Lumerical’s FDTD solver with the model shown in Fig. 1. It was assumed that all losses occurring within the intrinsic region of the device lead to the generation of electron–hole pairs (internal quantum efficiency of 1). The responsivity was calculated by

2 $$ R = \frac{1}{2}\int {{\upomega }\left| {{\varvec{E}}\left( {x,y,z,{\upomega }} \right)} \right|^{2} {\text{Im}}\left( {{\upvarepsilon }_{{\text{r}}} } \right)dV} \frac{e}{{\hbar \omega P_{{{\text{source}}}} }}, $$

with ***E*** being the electric field strength at a specific point within the intrinsic region. Here, ε_r_ is the relative permittivity including the vacuum permittivity, *ℏ* is reduced Planck’s constant, ω is the angular frequency, *e* is the elementary charge and *P*_source_ is the power of the incident wave front. The integration volume *dV* spans the intrinsic region of the NP by following the procedure from^25^. The wavelength-dependent refractive index of Ge *n*_Ge_(λ) used in simulation was extracted from ellipsometry measurements on the material used for diode fabrication (permittivity values were derived from this data as well; for data acquisition procedures see^26^).

The active layers of the Ge PIN photodiode were grown in a molecular beam epitaxy (MBE) system under coevaporation of the dopants. The deposition process on a p^–^-Si wafer started with 50 nm Si to smoothen the wafer surface followed by a 100 nm layer of Ge doped with a B concentration of 1·10^20^ cm^−3^. By curing this stack at > 850° C a virtual substrate formed which improved the quality of the subsequent Ge layers. The thickness of the p^+^-Ge layer was increased by another 300 nm of B-doped Ge. The intrinsic region has a thickness of 300 nm with a background doping of 1·10^14^ cm^−3^_._ The top *n*-region consists of 100 nm Ge and 100 nm of Si; each Sb-doped with a concentration of 1·10^20^ cm^−3^.

Device processing employed an inverted hard mask process to reduce the lateral dimensions and to improve structure quality of the electron beam lithography (EBL): First, the wafers with the MBE layers were coated with a SiO_2_ layer of 100 nm, then the NPs were defined through EBL, evaporation of Al_2_O_3_ and a lift-off process. A double dry-etching process first transferred the structure from the Al_2_O_3_ into the SiO_2_ hard mask and finally into the MBE layer stack. An SiO_2_ passivation layer was deposited using a plasma enhanced chemical vapor deposition process, then planarization by chemical mechanical polishing and selective back-etching of the planar surface was used to achieve a protrusion of the *n*-region of the NP-PD of approximately 50 nm out of the passivating oxide. The Al contact metallization was evaporated onto the sample under oblique incidence creating the crescent-shaped NA next to each NP in a self-aligned shadow mask process (Fig. 4b). As a consequence the length in *y*-direction of the NA is defined by *d*_NP_. The width *w* of the nanocrescent in *x*-direction follows from the protrusion of the *n*-region out of the passivation, and the angle of evaporation. For the sake of higher photocurrents the NP were arranged in a rectangular matrix with a pitch of Λ = 1000 nm covering an area *A*_m_. The SiO_2_ growth process and the subsequent chemical mechanical polishing step caused a metal roughness which suppressed plasmonic lattice effects.

The devices were characterized using an tunable light source with spectral power Φ(λ) with a line width of 5 nm. A semiconductor tester recorded the dark current *I*_d_ as well as the current from the illuminated device *I*_i_, which were used to calculate the optical responsivity *R*_*V*_ via

3 $$ R_{V} \left(\uplambda \right) = \left. {\frac{{I_{{\text{i}}} \left( V \right) - I_{{\text{d}}} \left( V \right)}}{\Phi }} \right|_{\uplambda } , $$

at external bias *V* and wavelength λ. Perpendicular incidence of the illumination was achieved by positioning a glass fiber above the arrays. The illuminated area was always smaller than the device area *A*_m_. The polarization was not tracked as the glass fiber does not maintain the state of polarization throughout the complete spectrum. Furthermore, the excitation source of the supercontinuum light source can lead to measurement artifacts at λ ∽ 1180 nm.

## Supplementary Information

Supplementary Information 1.

## Data Availability

All data generated or analysed during this study are included in this published article and its Supplementary Information files.
