# Supplementary material for: Photonic-plasmonic mode coupling in nanopillar Ge-on-Si PIN photodiodes
Source: Sci Rep. 2021 Mar 11;11:5723. doi: 10.1038/s41598-021-85012-z (PMC7952423; doi:10.1038/s41598-021-85012-z)
Supplement: Supplementary file 1 — Supplementary Information 1. [file 41598_2021_85012_MOESM1_ESM.pdf]

SUPPLEMENTARY INFORMATION TO

Photonic-Plasmonic Mode Coupling in Nanopillar Ge-on-Si PIN Photodiodes

Lion Augel[1,2*], Jon Schlipf[3], Sergej Bullert[2], Sebastian Bürzele[2],
Jörg Schulze[2], and Inga A. Fischer[2,3]

[1]Micro and Nano Systems, Brandenburg University of Technology Cottbus-Senftenberg, Cottbus 03046, Germany
[2]Institute of Semiconductor Engineering, University of Stuttgart, Stuttgart 70569, Germany
[3]Experimental Physics and Functional Materials, Brandenburg University of Technology Cottbus-Senftenberg, Cottbus 03046, Germany
*[*lion.augel@b-tu.de](mailto:lion.augel@b-tu.de)*

This file contains

1. Electric field profiles for parameter variations 2
   1.1. Variation of superstrate refractive index n_{sup} 2
   1.2. Variation of protrusion h 3
2. Electric device characteristic 4
3. Electro-optical device characteristic: Comparison with reference 5

1. Electric field profiles for parameter variations

In the following, we show simulation results for the electric field components $\boldsymbol{E}_i(x, y, z)$ $(i = \boldsymbol{x}, \boldsymbol{y}, \boldsymbol{z})$ in our devices under illumination with $\boldsymbol{E}_0 \| \boldsymbol{y}$.

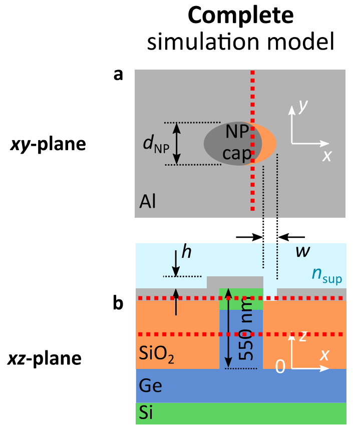

Supplementary Figure S1. Orientation of crosscuts (with reference to Fig. 1)

1.1. Variation of superstrate refractive index n_{sup}

By varying the refractive index on top of the device the plasmonic modes can be shifted. Due to their high field strength, the LSPR excited under illumination with $\boldsymbol{E}_0 \| \boldsymbol{y}$ tend to react much more strongly to changes within their surroundings.

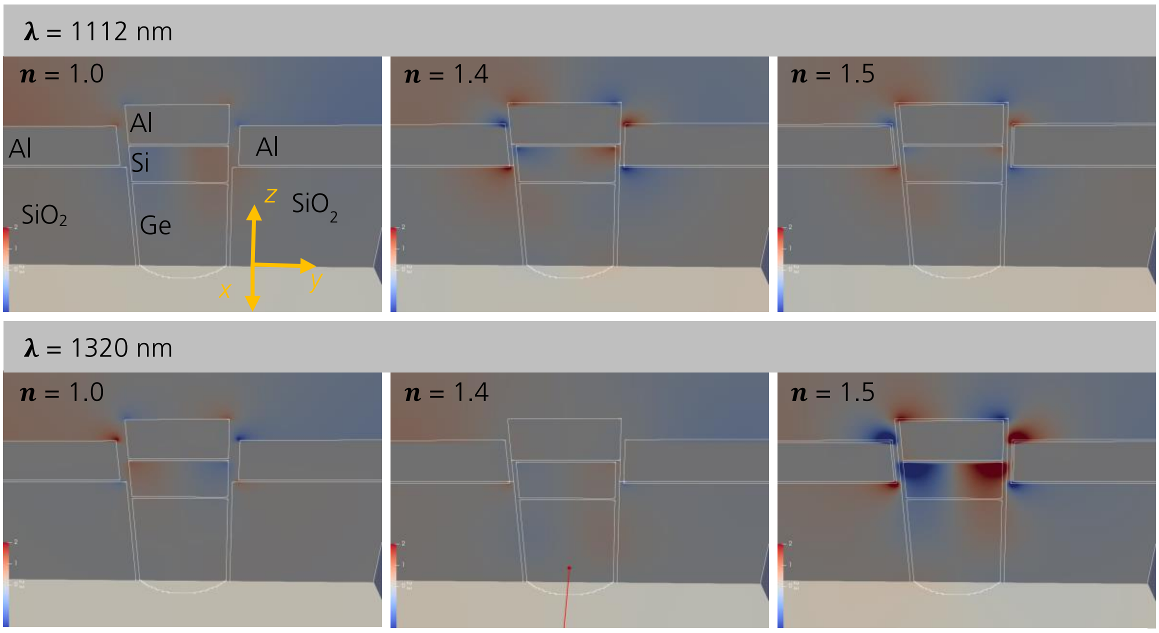

Supplementary Figure S2. Plots of $\boldsymbol{E}_z(x, y, z)$ for two different wavelengths with varying superstrate refractive index. All plots show a cross-sectional device image in direction of y under illumination with $\boldsymbol{E}_0 \| \boldsymbol{y}$. d_{NP} = 300 nm, h = 50 nm, w = 100 nm. Horizontal plane approximately 250 nm below the top Si-Ge interface.

1.2. Variation of protrusion h

Illumination under $\boldsymbol{E}_0 \| \boldsymbol{y}$ gives rise to localized surface plasmon resonances with a high field strength located within a small volume at the tip of the crescent nanoapertures.

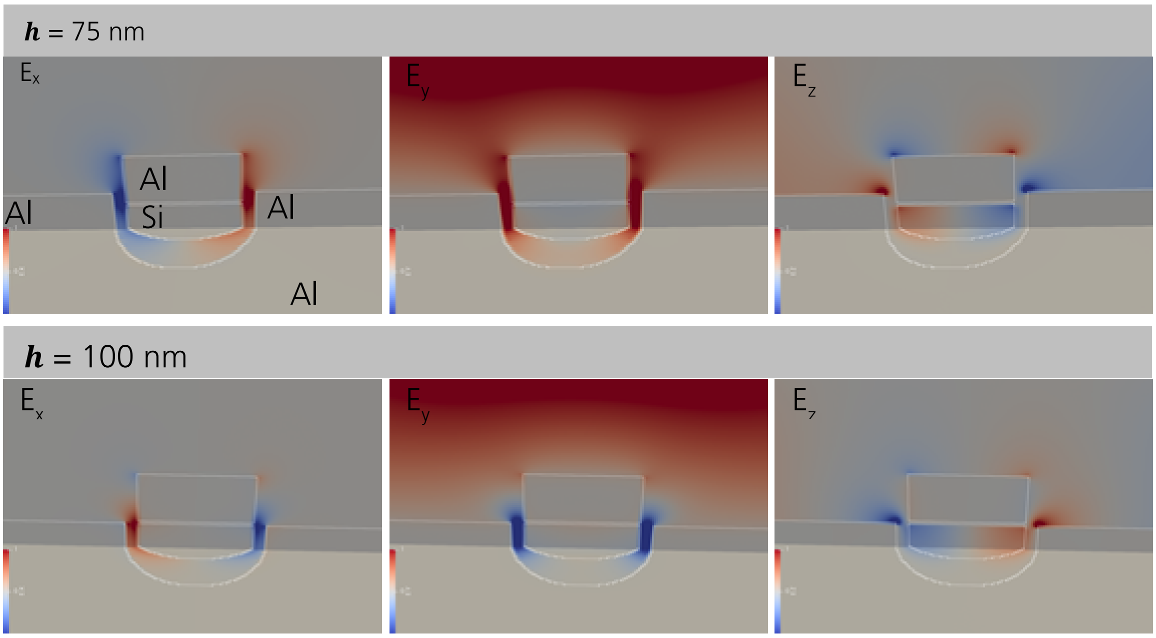

Supplementary Figure S3. Plots of $\boldsymbol{E}_i(x, y, z)$ with $i = \boldsymbol{x}, \boldsymbol{y}, \boldsymbol{z}$ for two different values for h. Cross-sectional device image in direction of y under illumination with $\boldsymbol{E}_0 \| \boldsymbol{y}$ at λ = 1483 nm. d_{NP} = 300 nm, w = 100 nm. Horizontal plane approximately 50 nm above the top Si-Ge interface.

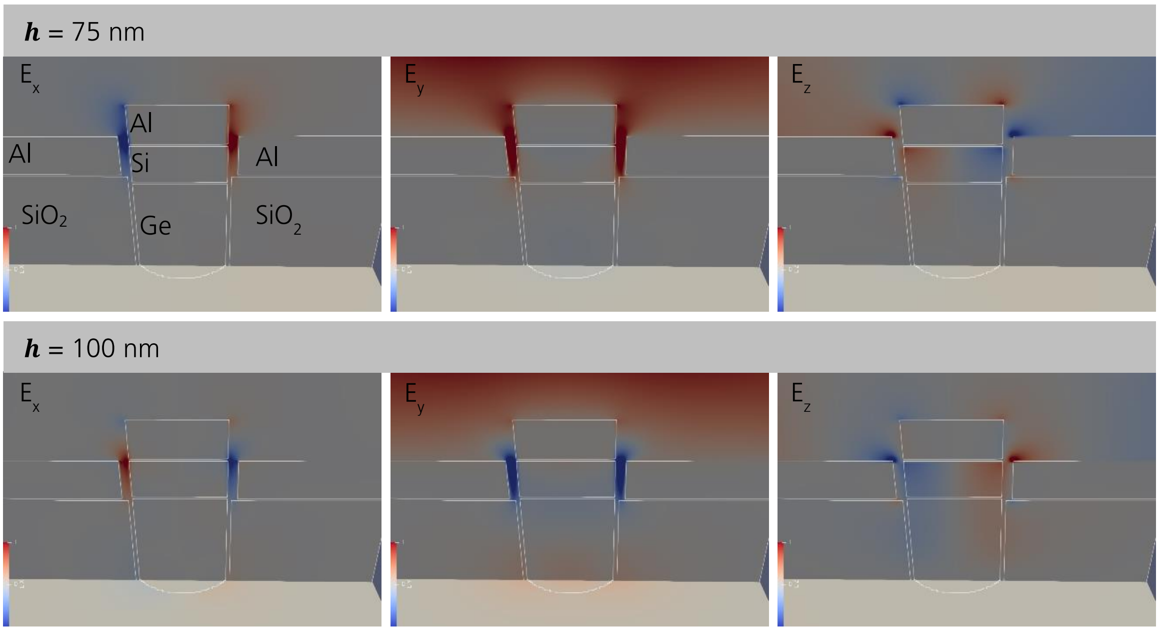

Supplementary Figure S4. Plots of $\boldsymbol{E}_i(x, y, z)$ with $i = \boldsymbol{x}, \boldsymbol{y}, \boldsymbol{z}$ for two different values for h. Cross-sectional device image in direction of y under illumination with $\boldsymbol{E}_0 \| \boldsymbol{y}$ at λ = 1483 nm. d_{NP} = 300 nm, w = 100 nm. Horizontal plane approximately 250 nm below the top Si-Ge interface.

2. Electric device characteristic

From the current-voltage characteristic $I(V)$ of a device the following figures of merit can be calculated through

$$I = I_s\left(e^{\frac{V_D}{\eta V_T}} - 1\right) = I_s\left(e^{\frac{V - R_S I}{\eta V_T}} - 1\right)$$

where I_s is the saturation current, V_D the voltage across the diode, V_T the thermal voltage, η the ideality factor and R_S the series resistance of the diode.

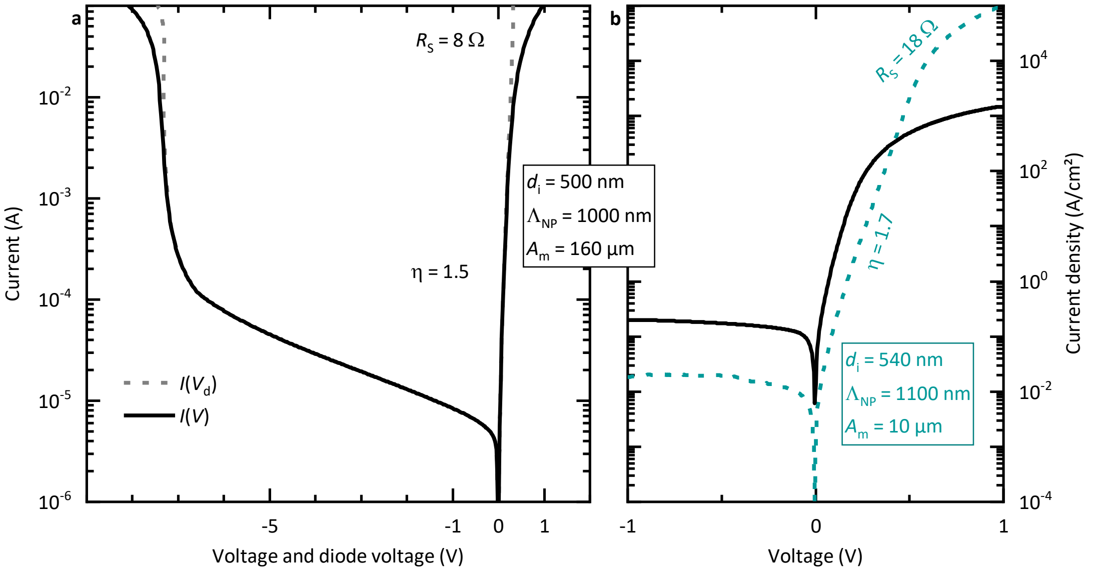

Supplementary Figure S5. (a) Electric characteristic of a device with diameter of A_m = 160 µm of the NP array. The measured current is plotted over the applied voltage V as well as the diode voltage V_d (where the series resistance has been eliminated). (b) Current density for the device from (a) in comparison to a different device with A_m = 10 µm.

3. Electro-optical device characteristic: Comparison with reference

Here, we provide the responsivity spectrum of a reference detector as baseline and compare it to the NP-PD responsivity spectra.

The reference detector is a (non-structured) bulk photodetector with an identical semiconductor layer stack. When measuring the reference detector responsivity spectrum under vertical incidence, however, the topmost Si layer can only be assumed to be transparent for incident wavelengths above ~1150 nm as a consequence of the indirect bandgap of Si. The extraction of the responsivity of Ge-on-Si photodetectors grown in the same MBE chamber and with a similar semiconductor layer structure except for individual layer thicknesses is discussed in detail in Ref. [S1]. The wavelength dependence of the responsivity R_{opt} of a photodetector is influenced by the wavelength-dependence of the external quantum efficiency $\eta(\lambda)$ [S2]:

$$R_{opt}(\lambda) = \eta(\lambda)\frac{q}{ch}\lambda,$$

where q is the elementary charge, c is the speed of light, h is the Planck constant and λ is the wavelength of the incident light. Here, we combine the measured spectrum of the reference detector in the wavelength range of 1210 – 1700 nm with a linear extrapolation of the responsivity to lower wavelengths, this approach is motivated by results obtained for similar devices (see Fig. 3.5 in Ref. [S1]). The scaled reference spectrum is also used as a baseline for peak fitting (Fig. 5) in order to consider contributions to the responsivity originating from a remaining planar Ge layer underneath the nanopillar structures (Fig. 1). The baseline is rescaled for each NP-PD device to take process variations into account.

Several competing effects can be expected to play a role when comparing the NP-PD responsivity to that of a reference device under identical illumination conditions, e.g.

- a reduction in the absorbing volume of Ge in the NP-PD photodetectors compared to bulk devices,
- an increase in absorption for NP-PD photodetectors compared to bulk devices due to the antireflective properties of the patterned surface as well as an increase in the effective penetration depth of the incident light as a result of structuring,
- a reduction in absorption for NP-PD photodetectors compared to bulk devices as a result of the metallic top layer that leaves only small apertures for light to impinge onto the Ge nanopillar structures,
- an increase in absorption for NP-PD photodetectors compared to bulk devices as a result of plasmonic enhancement sustained by the nanocrescent holes.

Instead of a detailed discussion of these effects, which exceeds the scope of this work, we provide two different comparisons of the NP-PD responsivity spectra with that of the reference device. The responsivity spectra as measured are shown in Fig. S6. Here, it can be seen that the responsivities of the NP-PD devices are drastically reduced compared to that of a reference detector.

In a second comparison we take into account the effects of a reduction in the absorbing volume of Ge in the NP-PD devices compared to a reference detector by normalizing to the actual available absorbing device volume as follows

$$R_{\mathrm{opt,norm}} = R_{\mathrm{opt}} \cdot \frac{\Lambda^2}{\pi d_{\mathrm{i}}^2}.$$

Here, Λ indicates the distance between two nanopillars in the square lattice, i.e. the lattice pitch, and d_i is the nanopillar diameter. The comparison of devices with different detector diameter indicates an enhancement in normalized responsivity for specific wavelength ranges (s. Fig. S7) which can exceed the responsivity spectrum of a non-structured device.

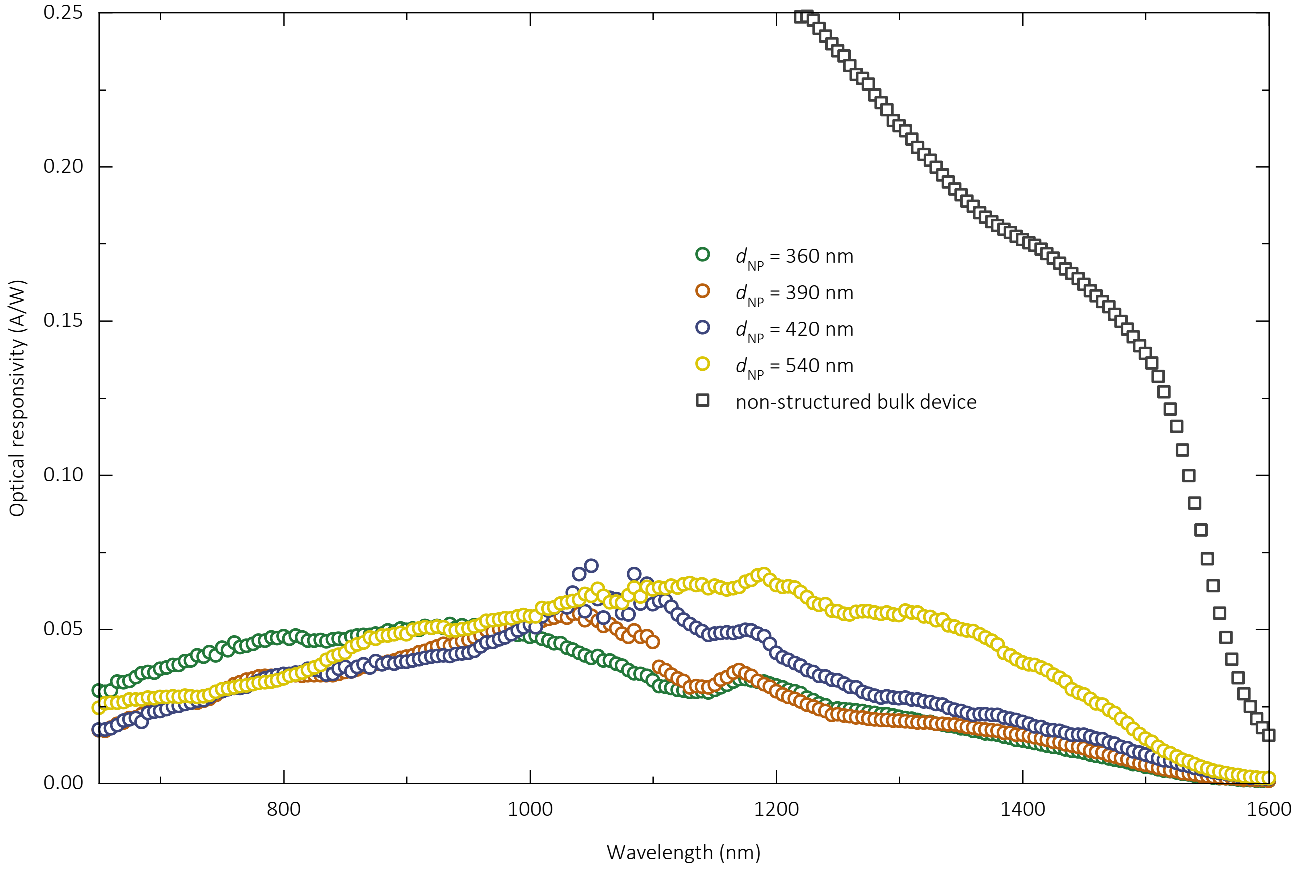

Supplementary Figure S6. Measured responsivity spectra of the nanopillar photodetector samples (○) in comparison to a non-structured bulk photodetector (□) with the same thickness of the absorbing layer and the same illuminated area.

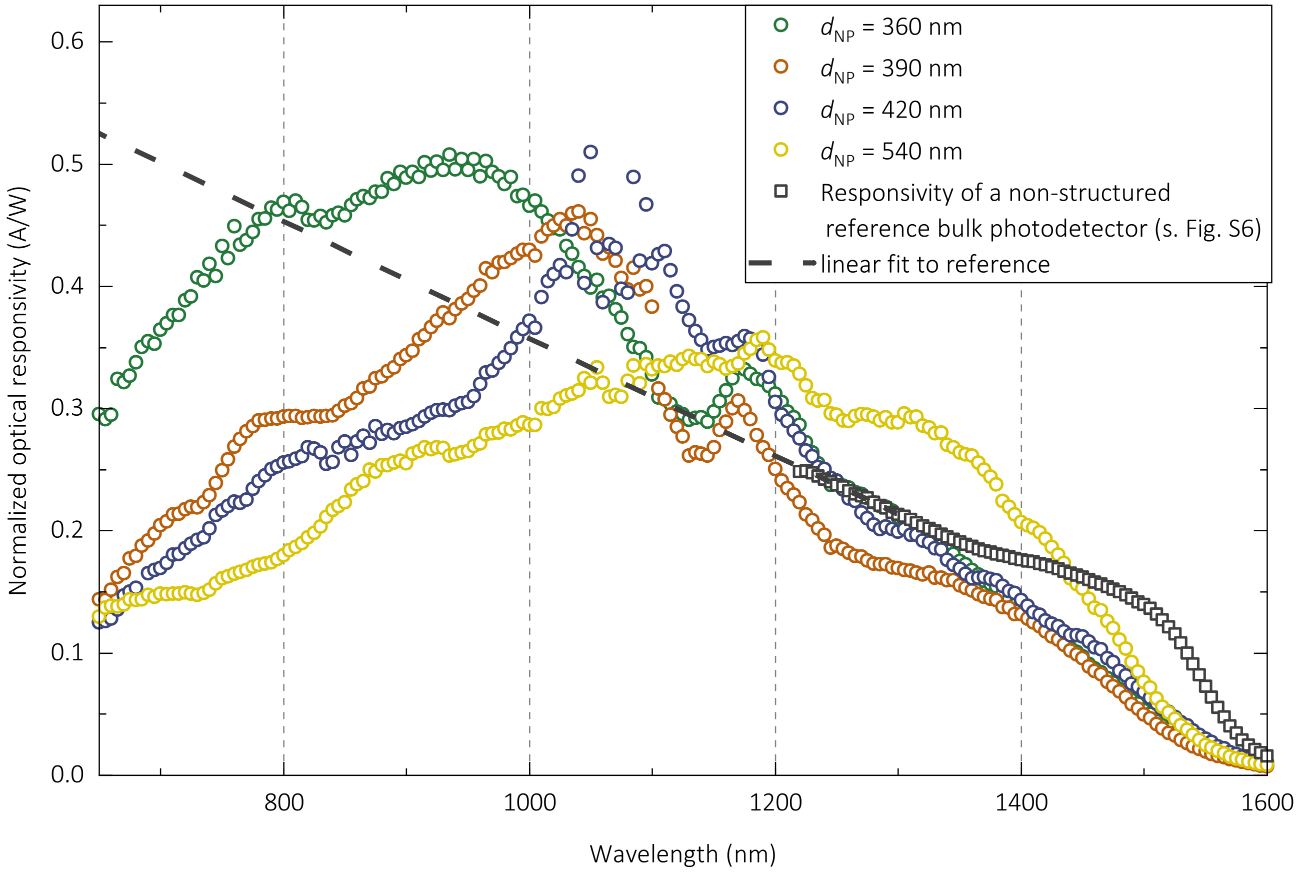

Supplementary Figure S7. Normalized responsivity spectra of the nanopillar photodetector samples (○) in comparison to a non-structured bulk photodetector (□) with the same thickness of the absorbing layer and the same illuminated area, which is used as reference. The linear fit (- - -) to the reference detector data for incident wavelengths between λ = 1210 nm and 1320 nm was used to extrapolate the baseline data down to λ = 650 nm.

References

[S1] Klinger, S. Germanium pin photodiodes on silicon and photonic integrated circuits: components for high-speed optical data communications, PhD thesis, Stuttgart, 2011. https://doi.org/10.18419/opus-2907

[S2] M. Jutzi, M. Berroth, G. Wohl, M. Oehme and E. Kasper, "Ge-on-Si vertical incidence photodiodes with 39-GHz bandwidth," in *IEEE Photonics Technology Letters*. 17 (7), 1510-1512 (2005), doi: 10.1109/LPT.2005.848546.
